# Evaluation of the Progression of Periodontitis with the Use of Neural Networks

**DOI:** 10.3390/jcm11164667

**Published:** 2022-08-10

**Authors:** Agata Ossowska, Aida Kusiak, Dariusz Świetlik

**Affiliations:** 1 Department of Periodontology and Oral Mucosa Diseases, Medical University of Gdansk, Orzeszkowej 18 St., 80-208 Gdansk, Poland; 2 Division of Biostatistics and Neural Networks, Medical University of Gdansk, Debinki 1 St., 80-211 Gdansk, Poland

**Keywords:** periodontist, periodontal diseases, diagnosis, computer simulation, artificial neural networks

## Abstract

Periodontitis is an inflammatory disease of the tissues surrounding the tooth that results in loss of periodontal attachment detected as clinical attachment loss (CAL). The mildest form of periodontal disease is gingivitis, which is a necessary condition for periodontitis development. We can distinguish also some modifying factors which have an influence on the rate of development of periodontitis from which the most important are smoking and poorly controlled diabetes. According to the new classification from 2017, we can identify four stages of periodontitis and three grades of periodontitis. Grades tell us about the periodontitis progression risk and may be helpful in treatment planning and motivating the patients. Artificial neural networks (ANN) are widely used in medicine and in dentistry as an additional tool to support clinicians in their work. In this paper, ANN was used to assess grades of periodontitis in the group of patients. Gender, age, nicotinism approximal plaque index (API), bleeding on probing (BoP), clinical attachment loss (CAL), and pocket depth (PD) were taken into consideration. There were no statistically significant differences in the clinical periodontal assessment in relation to the neural network assessment. Based on the definition of the sensitivity and specificity in medicine we obtained 85.7% and 80.0% as a correctly diagnosed and excluded disease, respectively. The quality of the neural network, defined as the percentage of correctly classified patients according to the grade of periodontitis was 84.2% for the training set. The percentage of incorrectly classified patients according to the grade of periodontitis was 15.8%. Artificial neural networks may be useful tool in everyday dental practice to assess the risk of periodontitis development however more studies are needed.

## 1. Introduction

Periodontitis is an inflammatory disease of the tissues surrounding the tooth that results in loss of periodontal attachment detected as clinical attachment loss (CAL). The supportive apparatus of the tooth (periodontium) consists of gingival tissue, alveolar bone, cementum, and periodontal ligaments. The mildest and most common form of periodontal disease is gingivitis which is the inflammation of the gingiva mostly caused by dental plaque. If the microbial biofilm is not removed properly within days or weeks, the changes in the gingiva start. The patient usually may notice bleeding, swelling, redness of the gingiva, and furthermore halitosis [1,2,3,4]. The common clinical signs of plaque-induced gingivitis include erythema, edema, bleeding, tenderness, and enlargement [5]. Potential modifying factors of plaque-induced gingivitis are hormonal changes, hyperglycemia, leukemia, smoking, malnutrition, prominent subgingival restorations margins, and hyposalivation [6,7,8]. Gingivitis is regarded as a necessary condition for subsequent periodontitis and progressive attachment loss. This process is highly connected with patient’s immune-inflammatory response which is why not all people with gingivitis will develop periodontitis [9,10].

The progression of periodontal disease depends on many factors and some patients will develop severe periodontitis in a short time whereas others can have a mild stadium for a whole life. Moreover, in some patients, periodontitis progression is less predictable than in others and involves different treatment plans. Well-known risk factors accelerating bone loss are nicotinism and poorly controlled diabetes, in addition to obesity, genetics physical activity, or nutrition. The clinician assesses the stadium of periodontitis taking into account the age of the patient which is an indirect way to evaluate the individual susceptibility to periodontitis. A common tool in assessing bone loss in daily practice is the measurement of the bone loss on radiograms expressed as a percentage of tooth length and divided by the age of the patient. In recent years, dentists compared clinical attachment loss (CAL) with the age of the patient to assess the standard clinical attachment loss for the age. This measurement can be completed by using the standardized probe UNC 15 [11,12].

Nowadays, artificial intelligence (AI) is becoming more important in medicine and in dentistry. It can be helpful in many fields where the human may be assisted and helped by new technologies. Machines are able to create algorithms through data learning, which allows them to handle prediction issues without human assistance. In a mathematical nonlinear model, neural networks (NNs) imitate the human brain by using artificial neurons that are similar to human neural networks. NNs can mimic human cognitive abilities including problem-solving and human thinking, which involves learning and making decisions. Simple neural networks contain three layers: input, hidden, and output. The input layer is where information enters the system, and the hidden layer is where the data are processed (where the system decides what to do). NNs may outline any input to output given a set of mathematical models if there is enough data, a lot of data [13].

## 2. Materials and Methods

### 2.1. Patients’ Population

This was a retrospective study, and the database consists of 110 patients, both genders aged 30 to 60 were included. The selection of the patients was performed in 2022 in the Department of Periodontology and Oral Mucosa Diseases, Medical University of Gdańsk. Only the patients with all necessary measurements were included into the study. There were 12 patients with stadium I periodontitis, 19 patients with stadium II periodontitis, 42 patients with stadium III periodontitis, 27 patients with stadium IV periodontitis and 10 patients with gingivitis. All groups included patients generally healthy or with diabetes or/and smokers. Patients with other systemic diseases and patients with dental implants were excluded. Dental assessment of the patients was performed, and the following indicators were included: gender, age, active nicotinism, the number of preserved teeth, approximal plaque index (API), bleeding on probing (BoP), pocket depth (PD), and clinical attachment loss (CAL). Patients were divided into 2 groups, the training group (90 persons) and test group (20 persons). Training group was the group in which neural networks learned how to classify patients and test group was a group in which the quality of neural networks was checked. 

### 2.2. Clinical Periodontal Measurements

Dental assessment of the patients was performed, and the following indicators were included: the number of teeth preserved, approximal plaque index (API), bleeding on probing (BoP), pocket depth (PD), and clinical attachment loss (CAL). The measurements were performed with the use of periodontal probe UNC 15 with a cylindrical shape, 15 mm scale, 1.75° cone taper, and 0.5 mm probe tip diameter [12].

According to the new classification in the context of the 2017 World Workshop on the Classification of Periodontal and Peri-Implant Diseases and Conditions, a patient with periodontitis has more than 2 interdental CAL in non-adjacent teeth or more than 2 teeth with buccal/oral CAL ≥ 3 mm and pocketing > 3 mm. From above criteria should be excluded:

- Teeth with gingival recession of traumatic origin;
- Dental caries near cervical area of the tooth;
- The presence of CAL at the distal surface of second molar due to the malposition
- Extraction of third molar;
- An endodontic lesion in the marginal periodontium;
- Vertical root fracture [11].

We can qualify patients with periodontitis into four stages to classify the severity and extent of the disease and to assess its complexity. Moreover, we can distinguish three grades to assess risk of progression and potential risk of systemic impact of the patient’s periodontitis [11].

Stage I periodontitis is the mildest form of periodontitis which develop just after the gingivitis. It is crucial to capture this stadium and implement the correct intervention and monitoring. Stage II periodontitis is moderate periodontitis with characteristic lesions in the periodontium. Professional management can arrest the disease’s progression. Stage III periodontitis is when the clinical attachment loss is more advanced and there is a risk for additional tooth loss. Stage IV periodontitis is a stage similar to stage III but also there is a need to complex dental rehabilitation due to the teeth loss, disabled masticatory function, and risk of loss the dentition Table 1.

The classification to the stage is mostly carried out on the basis of CAL and radiographic bone loss (RBL). If a stage shifting complexity factor(s) were eliminated by treatment, the stage should not be changed to a lower since the original stage complexity factor should always be taken into consideration [11].

Grading allows us to assess the progression of periodontitis and it is not dependent on staging. Each patient can have a different rate of progression of periodontitis. Due to the new classification, there is direct and indirect evidence of periodontitis progression Table 2. Direct evidence requires diagnostic radiographs from the past, and indirect evidence requires the assessment of the bone loss and taking into account the age of patient [14,15,16]. The new classification distinguishes 3 periodontitis grades A, B, and C. They can be modified by some risk factors such as smoking or the presence of poorly controlled diabetes. There is also a group of patients who is less responsive to the standard periodontal treatment due to some other risk factors for example genetics [17]. The aim of the grading assessment is to find the best treatment for periodontitis by taking into consideration the rate of progression. Bone loss in percentage divided by the age of the patient was used in the periodontal risk assessment (PRA) system [18].

Artificial intelligence (AI) is gaining importance in the fields of medicine and dentistry nowadays [13]. It can be useful in a variety of situations where new technologies might benefit and help people. AI can help us in the medical profession, particularly in areas such as radiology, pathomorphology, oncology, cardiology, psychiatry, nuclear medicine, and many more [19,20,21,22,23]. One way to comprehend the working of the nervous system, which we are unable to examine under natural conditions due to the limits of contemporary research techniques, is through the use of computer models of neural networks [24,25,26,27,28]. In silico methods have been widely used recently in a variety of contexts, including cancer, autoimmune, and neurodegeneration, to identify potential innovative pharmaceutical treatments [29,30,31].

### 2.3. Artificial Neural Network

The mathematical models that simulate how the brain works are called neural networks. It is a computing system made up of numerous processing components that are intricately integrated. It analyzes the data from outside sources and produces dynamic state responses. The ANN was created using a software network simulation program (Statistica Automated Neural Networks, TIBCO Software Inc. (Palo Alto, CA, USA, 2017). Statistica (data analysis software system), version 13. http://statistica.io, accessed on 1 September 2021). In order to obtain the best efficiency, one hundred multi-layer perception neural network models (MPL) were built. Neural network models were classified as classification networks, their task was to assign patients to one of the three classes related to the grading of periodontitis or to assess patients as healthy. In created models, the methods of “teacher projects” were used. The number of hidden neurons, the error function as a sum of squares or mutual entropy, and activation functions of hidden and output neurons from the available: linear, logistic, hyperbolic tangent, exponential, or sine were selected automatically.

#### 2.3.1. ANN Construction

**Figure 1:**
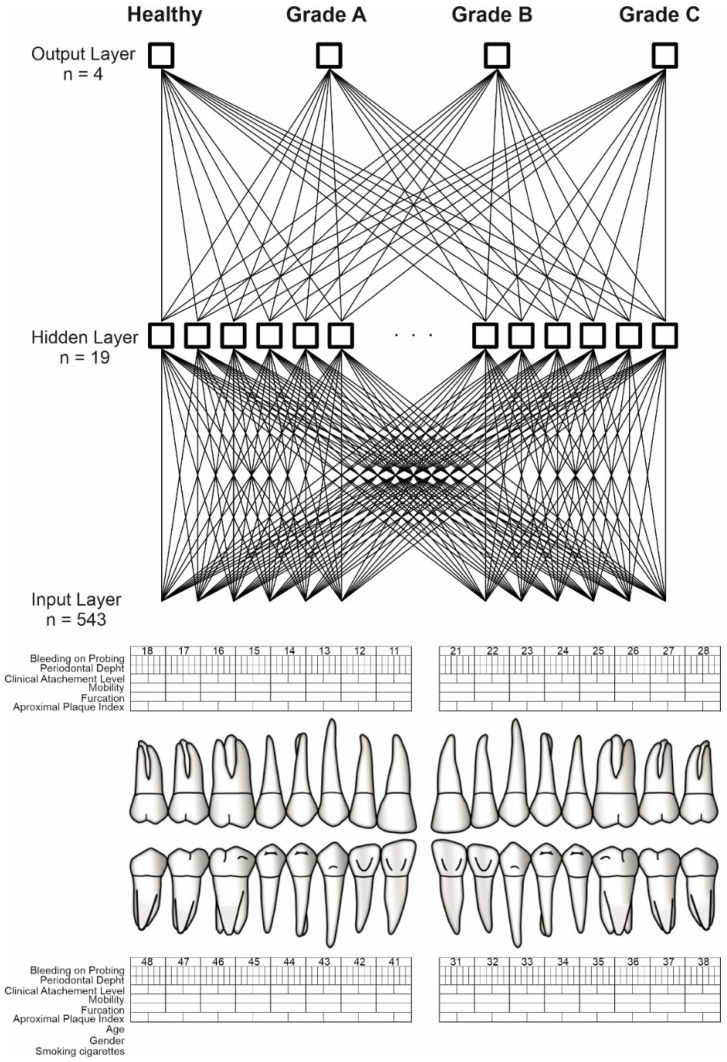
ANN construction. Source: https://www.periodontalchart-online.com/uk/ (accessed on 1 May 2020). Periodontal chart with input layer (n = 543), hidden layer (n = 19) and output layer (n = 4) which refers to periodontitis grading. Sex, age, smoking, approximal plaque index, bleeding on probing, periodontal pocket depth, and maximal interproximal loss of connective tissue attachment were all taken into account by the artificial neural network. For each patient, a set of 543 inputs was produced. By the use of the Statistica Automated Neural Networks, TIBCO Software Inc. (2017). Statistica (data analysis software system), version 13. http://statistica.io (accessed on 1 September 2021) the output layer has been received. The output layer consists of three grades (A,B,C) and a group of healthy patients.

Finally, the model of neural networks which was characterized by the lowest error and the highest quality of testing was selected. The neural network consisted of three layers: input, hidden, and output. The corresponding numbers of neurons in the individual layers were 543, 19, and 4 Figure 1. The activation and rejection levels for the neuron outputs were selected automatically by an artificial neural network simulator in order to minimize losses. During the learning process with the teacher, the weight connections between the neurons were modified by the BFGS algorithm [32]. The logistic activation function and Softmax were used for the neurons in the hidden layer and the output layer, respectively. The learning coefficient was 0.01 and the number of epochs was set to 1000, where the order of the presented cases for the neural network was different in each epoch. The initialization of weights for the neural network was performed randomly using Gaussian method. To calculate the classification quality of the artificial neural network, all patients were randomly divided into two groups: teaching and testing. The training group consisted of 90 patients and the testing group of 20 patients.

#### 2.3.2. Input Signals for Artificial Neural Network

The artificial neural network used information from patients’ records regarding sex, age, smoking, oral hygiene, periodontal pocket depth, and maximum interproximal loss of connective tissue attachment. A set of 543 inputs was prepared for each patient.

### 2.4. Software Simulation of ANN and the Statistical Analysis

All calculations were performed in the Statistica Automated Neural Networks, TIBCO Software Inc. (Palo Alto, CA, USA, 2017). Statistica (data analysis software system), version 13. http://statistica.io (accessed on 1 September 2021).

## 3. Results

### 3.1. Basic Characteristics

The study group of 110 patients included 9.1% of healthy subjects without periodontitis, 13.6% of patients with grade A, 39.1% with grade B, and 38.2% with grade C. Regarding the stages of periodontitis, in the study group 10.9% were patients with stage I, 17.3% with stage II, 38.2% with stage III and 24.5% with stage IV. The highest percentage of cigarette smokers was in the group of patients with stage C periodontitis at 50.0%. In the remaining groups, the percentage of smokers does not exceed 10% (Table 3). The average age of healthy volunteers was 33.1 (95% CI: 29.8–36.4), patients with grade A was 43.1 years (95% CI: 40.1–46.0), with grade B 48.1 years old (95% CI: 46.0–50.2) and with grade C 45.8 years (95% CI: 43.8–47.9). There were statistically significant age differences in relation to the grading of periodontitis (*p* < 0.0001). Healthy volunteers were significantly younger than patients with periodontitis (*p* < 0.05). The mean approximal plaque index (API) in the control group was 55.1% (95% CI: 35.8–74.5), in patients with grade A it was 64.7% (95% CI: 49.7–79.8), with grade B it was 78.5% (95% CI: 72.0–85.1) and with grade C it was 87.3% (95% CI: 81.6–93.0). There were statistically significant differences in the API oral hygiene status in relation to the groups (*p* = 0.0004). Patients with grade C had a significantly higher API index compared to healthy subjects (*p* = 0.0043) and patients with grade A (*p* = 0.0200). Average bleeding on probing index (BOP) in the control group was 40.3% (95% CI: 15.4–65.3), in patients with grade A it was 47.2% (95% CI: 33.2–61.1), with grade B it was 62.5 % (95% CI: 52.4–72.7) and with grade C it was 66.7 % (95% CI: 55.4–78.0). There were no significant statistical differences between groups (*p* = 0.0511). Average interproximal clinical attachment loss (CAL) in patients with grade A was 1.7 mm (95% CI: 1.0–2.4), with grade B 3.4 mm (95% CI: 2.8–3.9) and with grade C 4.6 mm (95% CI: 3.8–5.3). There were statistically significant differences in maximum CAL between groups (*p* = 0.0001). Patients with grade A had significantly lower scores than patients with grade B (*p* = 0.0143) and grade C (*p* < 0.0001).

The comparison of the demographic data of the training and test sets of the neural network is presented in Table 4. There were no statistically significant differences between the demographic factors in relation to the two sets of neural networks (*p* > 0.05).

On the other hand, the comparison of the periodontal assessment in the groups of the training and test sets of the neural network is shown in Table 5. There were no statistically significant differences in the periodontal assessment in relation to the two sets of the neural network (*p* > 0.05). 

### 3.2. Classification Assessment of the ANN

The quality of the neural network, defined as the percentage of correctly classified patients according to the grade of periodontitis was 84.2% for the training set. The percentage of incorrectly classified patients according to the grade of periodontitis was 15.8%. Detailed classification results of the neural network in the healthy group and in the groups of patients according to the grade of periodontitis from A to C are, respectively: 80.0%, 100.0%, 80.0%, and 80.0%. The corresponding percentages of incorrect classifications were, respectively: 20.0%, 0.0%, 20.0%, and 20.0%. The quality of the neural network for the women was: 90.9% and for the men 75.0%. On the other hand, the quality of the neural networks according to the grading and smoking was from 83.3% up to 100.0% (Table 6). Based on the definition of sensitivity and specificity in medicine we obtained 85.7% and 80.0% as correctly diagnosed and excluded diseases, respectively.

### 3.3. Sensitivity Analysis

Global sensitivity analysis gives us an idea of how important each network input field is. The values of the quotients for individual parameters range from 0.990 to 1.417 (Table 7).

### 3.4. Implementation of the Model into Clinical Practice

To use a previously saved neural network for new data, you need to open database. The active data file must contain the same fields as the input fields used to build the ANN model. During implementation, we are dealing with the new data, not those that were used to teach the ANN.

## 4. Discussion

Evaluation of the progression of periodontitis is an important step for a dentist while preparing a treatment plan, but also it might be helpful in motivating patients to participate actively in treatment. The above study takes also into consideration grades of periodontitis of the new classification of periodontitis from 2017. The parameters which were taken into consideration in the above study were age, gender, API, BoP, PPD, CAL, and smoking. The link between plaque (API) and periodontitis development is well known and the first works which take into consideration dental plaque as a causative factor of gingivitis and periodontitis are dated 1965. Due to not brushing, gingivitis will develop in all patients but periodontitis development is more complex, more diverse, and dependent on many factors [33,34,35]. According to Kornman et al. and Page et al. there has to be an interaction between the immune-inflammatory response of the host which depends on genetic polymorphism [36,37]. These polymorphisms together with environmental factors determine the development of disease [10,38,39,40]. The main parameter of gingivitis and periodontitis is bleeding on probing (BoP) which also differentiates both conditions into localized and generalized and tells us about the severity of inflammation. The high percentage of pockets depth of more than 6 mm also indicates higher severity [41]. Clinical attachment loss (CAL) to age ratio is the main parameter in grading and is the main indicator of the rate of progression. CAL higher than 6 mm means severe periodontitis but we should also take into consideration teeth that were already extracted and that will change our evaluation. Higher CAL at a younger age can be a predictor of fast-developing periodontitis [11]. Well-known unmodified risk factors for periodontitis are age, gender, and genetics, modified factors are bacteria, nicotinism, general diseases, malnutrition, socio-economic status, and stress [42]. Aging is a risk factor for periodontitis due to many causes from which we can highlight immunoaging which leads to increased susceptibility to infections because of the lower reactivity of lymphocytes. What is more, with the age the number of systemic diseases increases which may be an additional risk factor [41]. When it comes to gender men are at a greater risk for developing destructive periodontal disease than women because of sex-specific differences in immune function. Sex steroids, innate and acquired immunity, and higher levels of inflammatory cytokines, including interleukin-1β and tumor necrosis factor-α in men, makes them more susceptible to destructive periodontal disease [43]. The most important bacteria in periodontitis in dental plaque are Porphyromonas gingivalis, Tannerella forsythia, Treponema denticola, Aggregatibacter actinomycetemcomitans, and F. nucleatum [35,36,37]. One of the most important modified factors is nicotinism which increases the risk of periodontitis from 85% to 282%. According to the work by Dietrich et al., cigarette smoking is associated with a higher risk of tooth loss. The association between smoking and the incidence of tooth loss is stronger in men than women and stronger in younger than older individuals. Heavy smoking (≥15 cigarettes/d) was associated with >3 times higher risk of tooth loss in men [40,43,44]. The third major risk factor in periodontitis is poorly controlled diabetes which can increase the risk of periodontitis by 86%. Vascular changes caused by hyperglycemia are associated with the development of periodontal pathogens species. Moreover, diabetics show an exacerbated host response with hyperproduction of inflammatory mediators and polymorphonuclear dysfunction [45]. There are also some other risk factors of periodontitis that were not taken into consideration in the above. The artificial neural network may help clinicians to evaluate the risk of periodontitis development when taking into consideration modified and non-modified periodontitis risk factors. The overall accuracy of our ANN achieved 84.2% which is a similar result to other works where artificial neural networks were used. Thanathornwong et al. used a convolutional neural network (CNN) in their work to identify periodontally compromised teeth on digital panoramic radiographs. The average precision of these networks was 81% [46]. Lee et al. measured the radiographic alveolar bone level and assessed alveolar bone loss by using CNN. They made the diagnosis according to the new periodontitis classification from 2017. The accuracy of the case diagnosis was 85% [47]. Additionally, other authors used an artificial neural network to assess radiographic bone loss. Chang et al. used a deep learning hybrid method for staging periodontitis on panoramic radiographs. Deep learning was used to detect radiographic bone level or CEJ level and was used for periodontitis staging. The above method demonstrated high accuracy [48]. Krois et al. measured the percentage of periodontal bone loss using CNN. Neural networks showed at least a similar discrimination ability as dentists for assessing PBL on panoramic radiographs [49]. Özden et al. used three kinds of tools to classify periodontal disease. They used artificial neural networks, a supportive vector machine, and decision tree. The two last had better results than artificial neural networks [50]. In the study of Jun-Young Cha et al., the bone loss percentage was calculated and classified by convolutional neural networks on periapical radiographs and its accuracy was 88.89%. This method can be used to assess the severity of peri-implantitis [50,51,52].

In the work of Vadzyuk et al. two neural networks were designed with the dental examination, psychological testing, parameters of higher nervous activity, and heart rate variability analysis. The diagnostic sensitivity of the first prognostic model was 83.33% and the specificity was 92.31%. They concluded that assessment of the condition of teeth hard tissues, the level of oral hygiene, and the evaluation of psychophysiological features can effectively predict the risk of periodontal disease development in young people aged 18–23 [51]. 

According to the above study and the works of other authors, we can take into consideration artificial neural networks as a useful tool in daily practice. As it comes to periodontology ANN may be helpful in periodontitis grading assessment and periodontal treatment planning. The use of artificial neural networks may be helpful not only for periodontists but also for general dentists in evaluating the grades according to the new classification and it may facilitate communication between dentists of all specializations.

This work is limited by the relatively small number of patients. Further studies on a larger group of patients, taking into account risk factors are necessary.

## 5. Conclusions

Artificial neural networks may be a useful tool in everyday dental practice to assess the risk of periodontitis development. The accuracy of artificial intelligence is comparable to a dentist or even higher so it can be taken into consideration in clinical work as well as scientific work. Assessing the rate of progression of the periodontitis, especially in young people and at the initial stage of the disease might be sometimes difficult for the clinician, and additional tools such as artificial neural networks can ease the diagnosis and the treatment plan choice. Further studies are needed to improve this diagnostic method.

## Figures and Tables

**Table 1:** Periodontitis stage.

Periodontitis Stage		Stage I	Stage II	Stage III	Stage IV
Severity	Interdental CAL ^1^ at site of greatest loss	1–2 mm	3–4 mm	≥5 mm	≥5 mm
	Radiographic bone loss	<15%	15–33%	Extending to mid-third of the root or beyond	Extending to mid-third of the root or beyond
	Tooth loss	No tooths loss due to the periodontitis	No tooths loss due to the periodontitis	Tooth loss due to the periodontitis ≤ 4	Tooth loss due to the periodontitis ≥ 5
Complexity	Local	Probing depth ≤ 4 mm	Probing depth ≤ 5 mm	Probing depth ≥ 6 mm	Criteria as in III stage plus:
		Horizontal bone loss	Horizontal bone loss	Vertical bone loss ≥ 3 mm	Need for complex rehabilitation due to:
				Furcation II or III class	-masticatory dysfunction -secondary occlusal trauma
				Moderate ridge defect	-severe occlusal defect -less than 10 opposing pairs of teeth
Extent and distribution	Localized (<30% teeth involved), generalized, molar/incisor pattern				

^1^ clinical attachment loss.

**Table 2:** Periodontitis grade.

Periodontitis Grade			Grade A: Slow Progression	Grade B: Moderate Progression	Grade C: Rapid Progression
Primary criteria	Direct evidence of progression	Longitudinal data	Evidence of no loss over 5 years	<2 mm over 5 years	≥2 mm over 5 years
	Indirect evidence of progression	% Bone loss/age	<0.25	0.25 to 1.0	>1.0
		Phenotype	Heavy biofilm deposits and slow progression	Progression corresponding with biofilm deposits	Rapid progression which exceeds amount of biofilm, early onset of disease
Grade modifiers	Risk factors	Smoking	Non-smoker	<10 cigarettes/day	≥10 cigarettes/day
		Diabetes	Normoglycemic	Diabetes HbA1c < 7.0%	Diabetes HbA1c ≥ 7.0%

**Table 3:** Demographic characteristics.

	Healthy (n = 10)	A (n = 15)	B (n = 43)	C (n = 42)
Gender				
Female	6 (60.0%)	12 (80.0%)	30 (69.8%)	24 (57.1%)
Male	4 (40.0%)	3 (20.0%)	13 (30.2%)	18 (42.9%)
Grade				
gingivitis	10 (100.0%)	0 (0.0%)	0 (0.0%)	0 (0.0%)
1	0 (0.0%)	12 (80.0%)	0 (0.0%)	0 (0.0%)
2	0 (0.0%)	3 (20.0%)	12 (27.9%)	4 (9.5%)
3	0 (0.0%)	0 (0.0%)	28 (65.1%)	14 (33.3%)
Nicotinism	1 (10.0%)	1 (6.7%)	2 (4.7%)	21 (50.0%)
Age	33.1 (4.7)	43.1 (5.4)	48.1 (6.8)	45.8 (6.5)
API ^1^ (%)	55.1 (27.1)	64.7 (27.1)	78.5 (21.3)	87.3 (18.4)
BoP ^2^ (%)	40.3 (34.9)	47.2 (25.2)	62.5 (33.1)	66.7 (36.2)
PPD ^3^ (mm)	2.1 (0.1)	2.3 (0.1)	2.8 (0.5)	3.4 (0.9)
CAL ^4^ (mm)	-	1.7 (1.3)	3.4 (1.8)	4.6 (2.4)

^1^ approximal plaque index, ^2^ bleed-ing on probing, ^3^ pocket depth, ^4^ clinical attachment loss.

**Table 4:** Demographic factors for the training and the test groups.

	Training Group (n = 90)	Test Group (n = 20)	*p*-Value
Gender			0.5706
Female	60 (66.7%)	12 (60.0%)	
Male	30 (33.3%)	8 (40.0%)	
Age mean (SD)	45.5 (7.2)	43.9 (8.9)	0.3849

**Table 5:** Periodontal assessments for the training and the test groups.

	Training Group (n = 90)	Test Group (n = 20)	*p*-Value
API ^1^	79.8 (23.0)	69.2 (25.7)	0.0713
BoP ^2^	60.2 (35.0)	59.2 (31.6)	0.9051
PPD ^3^	2.9 (0.8)	2.7 (0.7)	0.1313
CAL ^4^	3.6 (2.2)	4.1 (2.2)	0.3952

^1^ approximal plaque index, ^2^ bleed-ing on probing, ^3^ pocket depth, ^4^ clinical attachment loss.

**Table 6:** The quality of the neural network according to the grades.

	Correctly%
All	84.2%
healthy	80.0%
A	100.0%
B	80.0%
C	80.0%
Gender	
Female	90.9%
Male	75.0%
Age (years)	
20–30	100.0%
30–40	80.0%
40–50	83.3%
50–60	85.7%
Cigarettes	
smoking	100.0%
no smoking	83.3%

**Table 7:** Global sensitivity analysis.

Parameter	Correctly%
Cigarettes	1.417
API ^1^	1.052
PPD ^2^	1.048
Age	1.038
CAL ^3^	1.015
Gender	1.0
BoP ^4^	0.994

^1^ approximal plaque index, ^2^ pocket depth, ^3^ clinical attachment loss, ^4^ bleed-ing on probing.

## Data Availability

All data generated or analyzed during this study are included in this published article.

## References

[1] Lang N.P., Bartold P.M. (2018). Periodontal health. J. Periodontol..

[2] Kinane D.F. (1999). Periodontitis modified by systemic factors. Ann. Periodontol..

[3] Zmora N., Bashiardes S., Levy M., Elinav E. (2017). The Role of the Immune System in Metabolic Health and Disease. Cell Metab..

[4] Quirynen M., Dadamio J., van den Velde S., de Smit M., Dekeyser C., van Tornout M., Vandekerckhove B. (2009). Characteristics of 2000 patients who visited a halitosis clinic. J. Clin. Periodontol..

[5] Tonetti M.S., Chapple I.L., Jepsen S., Sanz M. (2015). Primary and secondary prevention of periodontal and peri-implant diseases: Introduction to, and objectives of the 11th European Workshop on Periodontology consensus conference. J. Clin. Periodontol..

[6] Murakami S., Mealey B.L., Mariotti A., Chapple I.L.C. (2018). Dental plaque-induced gingival conditions. J. Periodontol..

[7] Kinane D.F., Attström R., European Workshop in Periodontology Group B (2005). Advances in the pathogenesis of periodontitis. Group B consensus report of the fifth European Workshop in Periodontology. J. Clin. Periodontol..

[8] Mariotti A. (1994). Sex steroid hormones and cell dynamics in the periodontium. Crit. Rev. Oral Biol. Med..

[9] Mariotti A., Mawhinney M. (2013). Endocrinology of sex steroid hormones and cell dynamics in the periodontium. Periodontology.

[10] Meyle J., Chapple I. (2015). Molecular aspects of the pathogenesis of periodontitis. Periodontol. 2000.

[11] Tonetti M.S., Greenwell H., Kornman K.S. (2018). Staging and grading of periodontitis: Framework and proposal of a new classification and case definition. J. Periodontol..

[12] Chapple I., Mealey B.L., Van Dyke T.E., Bartold P.M., Dommisch H., Eickholz P., Geisinger M.L., Genco R.J., Glogauer M., Goldstein M. (2018). Periodontal health and gingival diseases and conditions on an intact and a reduced periodontium: Consensus report of workgroup 1 of the 2017 World Workshop on the Classification of Periodontal and Peri-Implant Diseases and Conditions. J. Periodontol..

[13] Ossowska A., Kusiak A., Świetlik D. (2022). Artificial Intelligence in Dentistry—Narrative Review. Int. J. Environ. Res. Public Health.

[14] Papapanou P.N., Wennström J.L., Gröndahl K. (1989). A 10-year retrospective study of periodontal disease progression. J. Clin. Periodontol..

[15] Papapanou P.N. (1989). Patterns of alveolar bone loss in the assessment of periodontal treatment priorities. Swed. Dent. J. Suppl..

[16] Morelli T., Moss K.L., Preisser J.S., Beck J.D., Divaris K., Wu D., Offenbacher S. (2018). Periodontal profile classes predict periodontal disease progression and tooth loss. J. Periodontol..

[17] Tonetti M.S., Mombelli A. (1999). Early-onset periodontitis. Ann. Periodontol..

[18] Lang N.P., Tonetti M.S. (2003). Periodontal risk assessment (PRA) for patients in supportive periodontal therapy (SPT). Oral Health Prev. Dent..

[19] Kakileti S.T., Madhu H.J., Krishnan L., Manjunath G., Sampangi S., Ramprakash H. (2020). Observational Study to Evaluate the Clinical Efficacy of Thermalytix for Detecting Breast Cancer in Symptomatic and Asymptomatic Women. JCO Glob. Oncol..

[20] Fakhoury M. (2019). Artificial Intelligence in Psychiatry. Adv. Exp. Med. Biol..

[21] Feeny A.K., Chung M.K., Madabhushi A., Attia Z.I., Cikes M., Firouznia M., Friedman P.A., Kalscheur M.M., Kapa S., Narayan S.M. (2020). Artificial Intelligence and Machine Learning in Arrhythmias and Cardiac Electrophysiology. Circ. Arrhythm. Electrophysiol..

[22] Swietlik D., Bandurski T., Lass P. (2004). Artificial neural networks in nuclear medicine. Nucl. Med. Rev..

[23] Swietlik D., Białowas J. (2019). Application of Artificial Neural Networks to Identify Alzheimer’s Disease Using Cerebral Perfusion SPECT Data. Int. J. Environ. Res. Public Health.

[24] Świetlik D., Białowąs J., Kusiak A., Cichońska D. (2018). Memory and forgetting processes with the firing neuron model. Folia Morphol..

[25] Świetlik D. (2018). Simulations of Learning, Memory, and Forgetting Processes with Model of CA1 Region of the Hippocampus. Complexity.

[26] Świetlik D., Białowąs J., Kusiak A., Cichońska D. (2018). A computational simulation of long-term synaptic potentiation inducing protocol processes with model of CA3 hippocampal microcircuit. Folia Morphol..

[27] Świetlik D., Białowąs J., Moryś J., Kusiak A. (2019). Computer Model of Synapse Loss During an Alzheimer’s Disease-like Pathology in Hippocampal Subregions DG, CA3 and CA1—The Way to Chaos and Information Transfer. Entropy.

[28] Świetlik D., Białowąs J., Moryś J., Klejbor I., Kusiak A. (2019). Effects of Inducing Gamma Oscillations in Hippocampal Subregions DG, CA3, and CA1 on the Potential Alleviation of Alzheimer’s Disease-Related Pathology: Computer Modeling and Simulations. Entropy.

[29] Świetlik D., Białowąs J., Kusiak A., Krasny M. (2022). Virtual Therapy with the NMDA Antagonist Memantine in Hippocampal. Models of Moderate to Severe Alzheimer’s Disease, in Silico Trials. Pharmaceuticals.

[30] Świetlik D., Kusiak A., Ossowska A. (2022). Computational Modeling of Therapy with the NMDA Antagonist in Neurodegenerative Disease: Information Theory in the Mechanism of Action of Memantine. Int. J. Environ. Res. Public Health.

[31] Świetlik D., Kusiak A., Krasny M., Białowąs J. (2022). The Computer Simulation of Therapy with the NMDA Antagonist in Excitotoxic Neurodegeneration in an Alzheimer’s Disease—like Pathology. J. Clin. Med..

[32] Bishop C. (1995). Neural Networks for Pattern Recognition.

[33] Caton J.G., Armitage G., Berglundh T., Chapple I., Jepsen S., Kornman K.S., Mealey B.L., Papapanou P.N., Sanz M., Tonetti M.S. (2018). A new classification scheme for periodontal and peri-implant diseases and conditions–Introduction and key changes from the 1999 classification. J. Clin. Periodontol..

[34] Loe H., Theilade E., Jensen S.B. (1965). Experimental gingivitis in man. J. Periodontol..

[35] Löe H., Anerud A., Boysen H., Morrison E. (1986). Natural history of periodontal disease in man. Rapid, moderate and no loss of attachment in Sri Lankan laborers 14 to 46 years of age. J. Clin. Periodontol..

[36] Kornman K.S., Crane A., Wang H.Y., di Giovine F.S., Newman M.G., Pirk F.W., Wilson T.G., Higginbottom F.L., Duff G.W. (1997). The interleukin-1 genotype as a severity factor in adult periodontal disease. J. Clin. Periodontol..

[37] Page R.C., Kornman K.S. (2000). The pathogenesis of human periodontitis: An introduction. Periodontology.

[38] Papapanou P.N., Sanz M., Buduneli N., Dietrich T., Feres M., Fine D.H., Flemmig T.F., Garcia R., Giannobile W.V., Graziani F. (2018). Periodontitis: Consensus report of workgroup 2 of the 2017 World Workshop on the Classification of Periodontal and Peri-Implant Diseases and Conditions. J. Clin. Periodontol..

[39] Genco R.J., Borgnakke W.S. (2013). Risk factors for periodontal disease. Periodontol. 2000.

[40] López R., Smith P.C., Göstemeyer G., Schwendicke F. (2017). Ageing, dental caries and periodontal diseases. J. Clin. Periodontol..

[41] Shiau H.J., Reynolds M.A. (2010). Sex differences in destructive periodontal disease: Exploring the biologic basis. J. Periodontol..

[42] Leite F., Nascimento G.G., Scheutz F., López R. (2018). Effect of Smoking on Periodontitis: A Systematic Review and Meta-regression. Am. J. Prev. Med..

[43] Dietrich T., Walter C., Oluwagbemigun K., Bergmann M., Pischon T., Pischon N., Boeing H. (2015). Smoking, Smoking Cessation, and Risk of Tooth Loss: The EPIC-Potsdam Study. J. Dent. Res..

[44] Cairo F., Rotundo R., Frazzingaro G., Muzzi L., Pini Prato G.P. (2001). Il diabete mellito come fattore di rischio per la parodontite [Diabetes mellitus as a risk factor for periodontitis]. Minerva Stomatol..

[45] Thanathornwong B., Suebnukarn S. (2020). Automatic detection of periodontal compromised teeth in digital panoramic radiographs using faster regional convolutional neural networks. Imaging Sci. Dent..

[46] Lee C.T., Kabir T., Nelson J., Sheng S., Meng H.W., Van Dyke T.E., Walji M.F., Jiang X., Shams S. (2022). Use of the deep learning approach to measure alveolar bone level. J. Clin. Periodontol..

[47] Chang H.J., Lee S.J., Yong T.H., Shin N.Y., Jang B.G., Kim J.E., Huh K.H., Lee S.S., Heo M.S., Choi S.C. (2020). Deep Learning Hybrid Method to Automatically Diagnose Periodontal Bone Loss and Stage Periodontitis. Sci. Rep..

[48] Krois J., Ekert T., Meinhold L., Golla T., Kharbot B., Wittemeier A., Dörfer C., Schwendicke F. (2019). Deep Learning for the Radiographic Detection of Periodontal Bone Loss. Sci. Rep..

[49] Ozden F.O., Özgönenel O., Özden B., Aydogdu A. (2015). Diagnosis of periodontal diseases using different classification algorithms: A preliminary study. Niger. J. Clin. Pract..

[50] Cha J.Y., Yoon H.I., Yeo I.S., Huh K.H., Han J.S. (2021). Peri-Implant Bone Loss Measurement Using a Region-Based Convolutional Neural Network on Dental Periapical Radiographs. J. Clin. Med..

[51] Vadzyuk S., Boliuk Y., Luchynskyi M., Papinko I., Vadzyuk N. (2021). Prediction of the Development of Periodontal Disease. Proc. Shevchenko Sci. Soc. Med. Sci..

[52] Berglundh T., Armitage G., Araujo M.G., Avila-Ortiz G., Blanco J., Camargo P.M., Chen S., Cochran D., Derks J., Figuero E. (2018). Peri-implant diseases and conditions: Consensus report of workgroup 4 of the 2017 World Workshop on the Classification of Periodontal and Peri-Implant Diseases and Conditions. J. Periodontol..

